# UAV Block Geometry Design and Camera Calibration: A Simulation Study

**DOI:** 10.3390/s21186090

**Published:** 2021-09-11

**Authors:** Riccardo Roncella, Gianfranco Forlani

**Affiliations:** Department of Engineering and Architecture, University of Parma, 43124 Parma, Italy; gianfranco.forlani@unipr.it

**Keywords:** UAV, photogrammetry, camera calibration, GNSS-assisted block orientation, dome effect, Monte Carlo simulation

## Abstract

Acknowledged guidelines and standards such as those formerly governing project planning in analogue aerial photogrammetry are still missing in UAV photogrammetry. The reasons are many, from a great variety of projects goals to the number of parameters involved: camera features, flight plan design, block control and georeferencing options, Structure from Motion settings, etc. Above all, perhaps, stands camera calibration with the alternative between pre- and on-the-job approaches. In this paper we present a Monte Carlo simulation study where the accuracy estimation of camera parameters and tie points’ ground coordinates is evaluated as a function of various project parameters. A set of UAV (Unmanned Aerial Vehicle) synthetic photogrammetric blocks, built by varying terrain shape, surveyed area shape, block control (ground and aerial), strip type (longitudinal, cross and oblique), image observation and control data precision has been synthetically generated, overall considering 144 combinations in on-the-job self-calibration. Bias in ground coordinates (dome effect) due to inaccurate pre-calibration has also been investigated. Under the test scenario, the accuracy gap between different block configurations can be close to an order of magnitude. Oblique imaging is confirmed as key requisite in flat terrain, while ground control density is not. Aerial control by accurate camera station positions is overall more accurate and efficient than GCP in flat terrain.

## 1. Introduction

Accurate knowledge of camera interior orientation elements and proper mathematical modelling of the image formation process are key elements for image metrology. UAV photogrammetry is no exception in this respect [1]. Camera calibration, the process leading to the estimation of such model parameters, has long been (and still is) one of the most researched topics in close range photogrammetry [2] as well as in computer vision [3,4]. At least in the former area, there is general agreement on conditions providing optimal results [1,5]: camera parameters should be estimated in a Least Squares Bundle Block Adjustment (BBA) of a highly redundant camera network with strong geometry (highly convergent images, orthogonal roll angles, more than six rays per point, and large scale variations in images), a testfield with appropriate targets, highly accurate image matching of targets, image points covering full frame format, and significance tests to avoid over-parametrization [6,7,8]. Not all conditions need to be satisfied nor are Ground Control Points (GCP) generally necessary.

In the context of UAV camera calibration, assessing the accuracy of calibration parameters computed in various image block configurations by on-the-job self-calibration is still a disputed argument. Current technology also allows, besides the traditional case of block control by GCP, GNSS-assisted self-calibration. Evaluating the effects of residual calibration errors on tie point accuracy, in the case of pre-calibration as well as of on-the-job self-calibration, on the other hand, is of relevant interest, especially from a practical point of view.

In this paper a set of UAV synthetic photogrammetric blocks, built by varying terrain shape, surveyed area shape, block control (ground and aerial), strip type (longitudinal, cross and oblique), image observation, and control data precision has been synthetically generated. Through a set of Monte Carlo simulations the actual performance of each single configuration has been investigated. From an operational standpoint, analytical camera calibration comes in two versions: pre-calibration or on-the-job calibration. Both use a BBA with additional parameters; the former is normally executed in a laboratory test field under optimal camera network geometry, with estimated parameters kept fixed later in actual surveys; the latter estimates camera parameters as a by-product of the BBA of the actual survey block [9].

How to transfer close-range expertise on camera calibration, with its strong roots in industrial and metrology applications, to UAV photogrammetry is still an investigated topic. In a way, UAV photogrammetry is indeed a mix of close-range and aerial photogrammetry, as it inherits consumer cameras from the former and block geometry features from the latter (e.g., a basic flight plan made of nadir imagery along parallel strips). To complicate matters, UAV platforms come in two versions, fixed-wing and rotary-wing, with marked differences in flight management and camera pointing flexibility. The wealth of ongoing research devoted to UAV camera calibration witnesses a not-yet-settled issue, with many questions still open and even “old” certainties put under scrutiny [10].

Pre-calibration is well suited when the camera is mechanically stable and repeatable in focusing operations [11]; a further constraint is that it should be operated in the field under similar conditions (image scale, scene depth, etc.) to that of calibration. Most software packages provide specific camera calibration tools, with calibration patterns and automatic target detection to speed up operations. With fixed-wing platforms, cameras are easily removed from the drone body and so pre-calibration can take place in laboratory settings. With rotary-wing platforms both indoor and outdoor options are generally feasible. It should be noted, however, that if similarity of image scale between calibration and survey block is sought, indoor or laboratory calibration can be troublesome, especially with longer focal length optics.

As far as the alternative between pre-calibration and on-the-job calibration is concerned, the outcomes of the many study cases on UAV camera calibration are not all consistent, and the situation looks poised to remain so. The results of [12] found that, with dense ground control, differences between on-the-job and pre-calibration were not substantial. In [13], proper distortion modelling is the goal to pursue to avoid systematic errors; pre-calibration is recommended together with an after-flight calibration check based on k1-k2 parameters’ equifinality. Oblique imaging in the range of 20° to 45° with respect to nadir amounting to at least 10% of block images should be included to reduce doming. The authors of [14] recommend robust pre-calibration (longitudinal and double cross with a few oblique ones) and claim that an on-site block as small as 20 images, with four oblique images at block corners, in a scene with sufficient height variations, might be enough to achieve this aim. Additionally, using pre-calibrated parameters, they found virtually the same residuals on GCP for two flights executed at a three-day distance over the same test field, implying a good short-term stability of camera parameters. In a rectangular block with high-overlap nadir imagery, [15] found pre-calibration to be more accurate than on-the-job calibration, though the main improvement came from accurate camera distortion modelling. On the other hand, it has been found in empirical tests [16,17,18] that Interior Orientation (IO) elements are not stable or that the reliability of the pre-computed parameters is questionable, due perhaps to poor repeatability of focusing, shocks in landing or different ambient temperatures. According to [1], pre-calibration remains the best option in the case that basic conditions for self-calibration cannot be met on site. However, in practice, on-the-job calibration is the method of choice, perhaps optimizing flight parameters to meet both survey requirements and safe conditions for self-calibration.

The progress in feature-based matching, with tens of thousands of tie points extracted and often matched across more than a dozen images, makes self-calibration without targets possible [19,20], on condition of a reasonably textured scene. Therefore, tie points’ distribution over the full frame format and accurate image matching can be taken for granted in most survey flights. In his analysis, [1] highlights the importance of scale changes within images as a key factor allowing, even with limited geometric block strength, full or partial recovery of IO and distortion parameters. Flight planning software for UAVs commonly incorporates the so-called double-grid option, with cross strips providing the orthogonal roll angles to reduce projective coupling between Exterior Orientation (EO) and IO parameters. Multi-scale self-calibration, with scale changes between images arising from blocks flown at different altitudes, has been shown [14] being less effective, at least unless GCP are introduced [10,21].

Simulations, as well as empirical studies, showed that large systematic elevation errors (the so-called doming effect) could arise from inaccurate estimations of calibration parameters [22]. The addition of oblique imaging to nadir imagery along parallel strips has been proposed and shown to be beneficial [13,23,24,25]. Rather than adding another flight layer, even flying the longitudinal strips with moderate-to-strong (30° to 45°) camera axis inclination along flight direction [12,21,26] proved effective in eschewing systematic errors in elevation. The effectiveness of the gently oblique (20° camera pitch) double grid proposed by [26] has also been confirmed by [27]. More radically, the very advantage of using nadir images at all, as well as of the large overlaps of UAV blocks, has been questioned: from homologous ray intersection analysis, [10] suggests switching, whenever feasible, to a simple or double grid image acquisition mode where the UAV camera always points towards the center of the area of interest at ground level. On the other hand, a simulation study [28] showed that, with only gently inclined camera axes, otherwise negligible correlations among decentring and radial distortion parameters may arise and affect calibration results as well as reduce the doming effect mitigation of oblique imaging.

Most flight planning software allows for simple and double grid schemes and, for multi rotors, for Point Of Interest (POI) mode, where the UAV takes a circular path around a ground target that is always kept centred in the camera frame. It should be noted, however, that (to the best of authors’ knowledge) all experimental studies with oblique imaging have been performed with multi-rotor platforms, where the camera is normally mounted on a gimbal. Oblique imaging with fixed wings, though an option available in some platforms, is more difficult to achieve in practice, so meeting optimal conditions for on-the-job self-calibration with these platforms may be harder; [24] suggest including gently banked turns in the flight plan to this aim.

In aerial blocks, the basic camera network geometry is determined by image overlap (side and forward), as the area of interest is typically covered by nadir imagery along parallel strips. Increasing overlap to a much higher degree than necessary for stereo coverage is common in UAV blocks; due to high repeatability of extracted key points, it increases ray multiplicity and so network strength. How effective this larger overlap is in improving self-calibration is, however, questioned [10], as the average ray intersection angle decreases with increasing overlap.

In aerial and UAV photogrammetry, block georeferencing and block control by GCP are intertwined and enforced in the BBA. Finding rules for determining the most efficient density and distribution of GCP in a UAV survey is not a trivial task, given the number of parameters involved. Indeed, the topic is still a debated subject of investigation [29,30] and is further complicated if accurate camera station positions are employed. Using Camera Stations (CS) determined by on-board Global Navigation Satellite System (GNSS) receivers to georeference and control the block is indeed a more than 30-year-old technique [31], known as GPS-supported or GPS-assisted aerial triangulation [32,33]. In many of today’s papers this technique is (improperly, in the author’s opinion) referred to as Direct Georeferencing (DG), a term that should be restricted to blocks where camera E.O. data are all determined by GNSS-assisted inertial navigation, and in principle there is no need for tie points. The availability on the market of both fixed-wing and multi-rotor platforms equipped with dual frequency GNSS receivers with Real Time Kinematic (RTK) technology enables GNSS-assisted block georeferencing and control, minimising the need for control at ground level [34,35]. As this technology becomes less expensive and satellite constellations improve their coverage, ensuring cm-level accuracy, it can be expected that it will gain ground, especially whenever site conditions make GCP survey difficult [36,37]. Notice that RTK is not strictly necessary, though it allows quick, on-site checking of the positioning quality. Indeed, the GNSS observations might as well be recorded on board and elaborated later in Post Processing Kinematic (PPK) mode, exploiting more sophisticated processing options and possibly improving positioning accuracy [35,38].

Agreeing with the Computer Vision approach, [13] believe that GCP or GNSS-determined CS need not to be involved in the BBA but instead used to compute an Helmert transformation from the BBA arbitrary reference frame and the mapping reference frame. However, it is also acknowledged in the paper that GCP or GNSS-determined CS help to refine calibration or limit block deformations that may arise from un-modelled systematic errors (such as residual calibration errors) and, to some extent, might also improve calibration parameter estimation. It is therefore worth investigating whether moving the control from ground points to CS changes the accuracy of the calibration parameters in a self-calibrating BBA. A few experiences [11] as well as previous simulation studies [13,39] suggest camera calibration with UAV blocks flown with GNSS-assisted block georeferencing and control deserves a more systematic investigation. In particular, in early tests [34,40] and later ones [35] it has consistently been found that in nadir-only imagery blocks a bias in elevation could arise using self-calibration and that a way to cope with this problem is to use at least one GCP. Lately, however, no need for such single GCPs has been found if oblique images are added [27].

In the context of UAV camera calibration, this paper therefore has two objectives. The main one is to assess the accuracy of calibration parameters computed in various image block configurations by on-the-job self-calibration under realistic conditions, representative of two widespread operating scenarios in UAV surveys. Besides the traditional case of block control by GCP, a well-searched topic, of special interest in authors’ view is the performance of a GNSS-assisted self-calibrating BBA as a function of the number of GCP; more precisely, just one at block centre or none at all.

The second paper goal is to assess the effects of residual calibration errors on tie point accuracy in case of pre-calibration as well as of on-the-job self-calibration, again as a function of different block configurations.

Compared to other papers on the subject, the experiments herein try for a more systematic approach through simulations, to gain insight on the influence of several factors affecting UAV camera calibration. To this aim, a set of synthetic UAV photogrammetric blocks has been generated that encompasses overall 144 different combinations of landform, surveyed area shape, block control type (ground and aerial), number and type of strip layers, precision of image coordinates and control data. In a Monte Carlo (MC) scheme, each simulated block combination has been adjusted by a self-calibrating BBA where the simulated, true values of image and control data have been corrupted with random errors, executing 1000 runs for each combination. A similar approach, here applied in a more comprehensive test setup, has been already proposed by [41,42], applied to GNSS-assisted block orientation by [39] and also adopted by [35] to generate precision maps. Another example of a Monte Carlo simulation study focused on the dome effect is also presented in [43].

Of course, the problem dimensionality is so large that many other factors could have been considered in the simulations (first of all image overlap, instead kept fixed to values frequently adopted in today’s UAV surveys). A choice was made to limit computing time and memory storage.

## 2. Materials and Methods

For the simulated blocks to be as realistic as possible, it has been decided to build them from the BBA output of two real blocks, each flown over a different landform according to the same flight plan. The motivation for this choice is to avoid an unrealistic distribution of the tie points over a regular grid and, most of all, an artificially high and fairly homogeneous distribution of the tie point ray multiplicity i.e., of the number of images an object point is observed on. As the two sites present rather different characteristics, the tie point distribution and their multiplicity can be expected to differ as well; this should help in clarifying whether and how these two factors affect the calibration accuracy. In the following, first the characteristics of the real blocks are described, then the procedure to build the synthetic blocks is illustrated.

### 2.1. Characteristics of the Two Real Survey Flights

**Figure 1:**
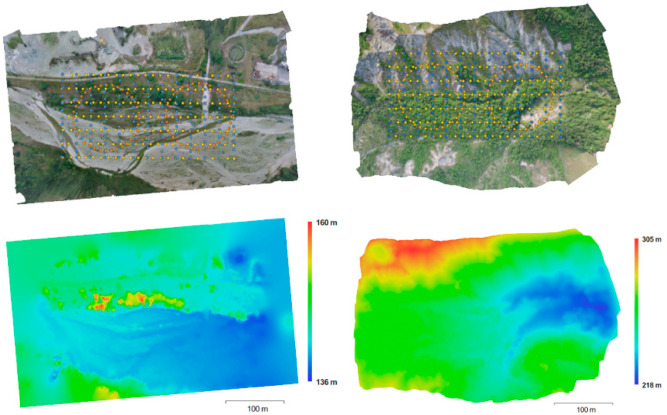
The top row shows the orthophoto of the Flat (**left**) and Hilly (**right**) areas, with superimposed the camera stations locations, colour-coded per strip type: longitudinal (yellow), cross (blue), oblique (orange). The bottom row shows the DEM generated from the sparse tie points.

**Figure 2:**
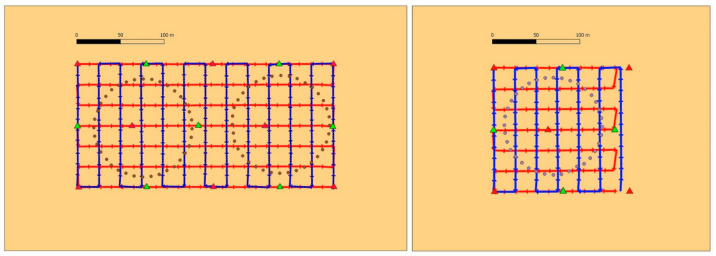
The surveyed area (orange background) with the trajectories of the three strip types: Longitudinal (blue); Cross (red); POI (brown). The figure depicts a Control type GCP with Enhanced control tightness (see Table 3) where GCP are represented by triangles. Different colours of triangles refer to control tightness: Basic (red) or Enhanced (red + green). (**Left**): LCO block configuration. (**Right**): HLCO block configuration.

The first block (Flat) images the Torrente Baganza riverbed (44°43′3″ N, 10°14′18″ E) made of bare terrain (gravel and sand) with bushes and a few rows of high trees. The second block (Hilly) images a steep ravine located in the Appenines, about 20 km South-West of Parma (Italy) (44°40′29″ N, 10°8′57″ E) with bare terrain, boulders as well as trees, grass and bushes. Images have been acquired with a DJI (Shenzhen, China) Phantom 3 equipped with a FC300X camera with a resolution of 4000 × 3000 pixels, a pixel size of 1.56 micrometres and a 3.61 mm nominal focal length (21 mm equivalent 35 mm format focal length). The flight plan (see Figure 1 and Figure 2) is made of three different strip types, all flown at constant elevation above sea level (a.s.l.), i.e., with nominal camera station positions all in the same horizontal plane:

- 7 nadir-imaging longitudinal strips with 80% forward overlap and 70% sidelap;
- 12 nadir-imaging cross strips with 80% forward overlap and 70% sidelap matching the longitudinal strips;
- 2 rings of 36 oblique images, regularly spaced along a horizontal circle, with camera axes pointing downwards at the circle centre ground projection (POI mode), with an angle from nadir close to 49 degrees. As the longitudinal strips length is designed to be twice the block width, the centre of each ring has been designed to be close to the (square) half-block centre while the circle radius is slightly larger than half the half-block diagonal.

Pix4D capture flight planning software has been used with the double grid option for shooting the longitudinal and cross strips as well as the ring of oblique images. Two separate flights have been executed in each site, one for the double grid, the other for the rings. The flight elevation above ground level (a.g.l.) is computed with respect to the lowest terrain point in Flat block and to the highest in Hilly block. Both full blocks (i.e., including all images of all strip types) have been oriented with Agisoft’s Metashape v. 1.5.3 and georeferenced on navigation data only. Figure 1 shows the orthophotos (top) and the DEMs (bottom) of both areas. The camera stations are shown, color-coded according to strip type, superimposed to the orthophotos. Table 1 summarizes the main characteristics of the two flights, that show different average GSD, number of extracted tie points, average image overlap and reprojection error.

On-the-job self-calibration has been executed in the BBA, enabling the estimation of the camera parameters listed in Table 2. Notice that the camera mount of the Phantom 3 is such that the largest side of the sensor is perpendicular to flight direction. As such, the Y axis is “along strip” and the X axis is “across strip”: the image coordinate system is oriented with the Y axis in flight direction and the X axis 90° clockwise with respect to Y.

No particular refinement of the BBA adjustment results has been carried out, as the goal of the operation was simply to provide data for the simulations.

### 2.2. Generation of Block Configurations for the Simulation

Different block configurations are generated by selectively removing from the full block one of the strip types (namely: the cross strips, the oblique images or both) in the original projects. Each block configuration is labeled according to the strip type it contains using the letters L, C and O to label longitudinal strips, cross strips and POI images, respectively. For instance, an LCO configuration corresponds to the original full block, while an LC configuration represents a block made of longitudinal and cross strips only, and so on. A block configuration is therefore made of one, two or three different strip types.

In order to account for the effect of different area shapes on calibration, exploiting the 1:2 width-to-heigth ratio of the rectangular original block, the second half of the original full block (LCO) has been cut out, allowing the generation of square block configurations also from the (original) first half-block. Configurations derived from this square block recieve the prefix H. As such, the HLO configuration is made of a longitudinal square block complemented with a ring of oblique images; HO is a POI (single ring) over the square area, and so on. For each block configuration, camera stations and pose, tie point ground coordinates and camera calibration parameters are exported to act as true data for the simulation.

Overall, four block configurations (LCO, LC, LO and L) have been considered for the rectangular area and five (HLCO, HLC, HLO, HL and HO) for the square area. Each configuration has been generated for both the Flat and Hilly areas.

As far as block control is concerned, both ground-only (GCP case) and GNSS-assisted (GNSS case) have been tested with both Basic and Enhanced tightness (see Table 3). In GCP Basic (see red triangles in Figure 2), 8 and 5 GCP are placed at the corners and in the middle of the block square(s), respectively, for the rectangular and square area. In GCP Enhanced, 15 and 9 GCP are arranged in three rows along the longitudinal flight lines, respectively, for the rectangular and square blocks (see red and green triangles in Figure 2). In GNSS Basic as well as in GNSS Enhanced, all camera stations’ positions are used as control information; however, in the former no additional GCP are used, while in the latter a single GCP, located at the block centre, is fixed.

**Table 3:** The two block control cases each with two different tightness levels, depending on the number of GCP fixed.

Block Control Case	Tightness
GCP (Ground)	Basic: 5–8 GCP
	Enhanced: 9–15 GCP
GNSS (Aerial)	Basic: no GCP
	Enhanced: 1 GCP

Two sets of observation precisions have been considered to simulate both a medium as well as a high-precision data set (see Table 4). Though “average” users cannot do much to improve the precision of the control data, in principle a better quality of positioning data can be foreseen. As far as the GNSS case is concerned, a better hardware (especially a better antenna), a good satellite configuration and expertise in PPK GNSS data might do the job. As far as the GCP case is concerned, using a Total Station shifts the accuracy range below the cm level [30]. Tie point image coordinate precisions depend on image quality and object texture characteristics so, once the image block is acquired, the user has a limited ability to intervene. Some differences can be expected in point identification performances, if different Structure from Motion algorithms (i.e., different software packages) are used, or if different processing parameters are chosen (e.g., the Orientation quality parameter in Metashape). A matching precision of 1 pixel and of 1/3rd of a pixel has been considered.

Table 5 summarizes the combinations of BBA configurations tested in the MC simulation. The combination of Area shape and Strip types yields nine different configurations. Combining them in all possible ways (16) with the parameters Landform, Measurement precision, Block control type and Block control tightness, a total of 144 different cases were investigated in the MC simulations.

### 2.3. Generation of True Values and True Errors for the Synthetic Data

The true values of the exterior and interior orientation parameters (including the camera optical and sensor distortion parameters) and of the tie points’ ground coordinates of the simulated blocks are taken from the real blocks, i.e., from the estimated parameters values after a free-net self-calibrating BBA executed with Agisoft MetaShape (Agisoft, St. Petersburg, Russia) on the two real blocks. The tie points’ distribution and their multiplicity in the simulated block is also taken from the real blocks. To this aim, the list of tie points in each image has been exported from MetaShape and the tie point image coordinates’ true values have been generated by projecting the ground point coordinates with the collinearity equations, according to the estimated exterior orientation parameters and camera parameters. The synthetic image coordinates so obtained incorporate the optical and sensor frame distortion estimated for the real block. Therefore, though the same camera has been used in both real flights, the synthetic block’s IO parameters are slightly different. For instance, the focal length true value for the Flat terrain block is 2335 pixels (21.01 mm equivalent 35 mm format focal length) while for the Hilly terrain block it is 2320 pixels (20.88 mm equivalent 35 mm format focal length). Normally distributed errors with standard deviations according to Table 4 have been generated in each run of the MC simulation and added to the true observations.

Running the BBA in the MC simulations, the standard deviations assigned to the observations should be the same as those reported in Table 4. This is true for the tie points’ image coordinates; for the GCP and the CS coordinates, however, in a real block it might be advisable to reduce their standard deviations (i.e., by a factor three/four) to reduce unmodeled errors, as the much larger number of image observations with respect to the other observation types needs to be counterbalanced by increasing the weights of the latter [1,40,42,44]. In this case, however, being the observations affected by zero mean gaussian errors, the effect of varying to some extent the weights of the observations have negligible effects on the final results.

### 2.4. Accuracy Evaluation of Camera Calibration Parameters

The calibration parameters’ accuracy will be investigated at the single parameter level as well as at a global (image) level. The former analysis will focus primarily on the three IO parameters, the latter on the largest residual distortion over the whole image frame.

Correlations between parameters play a key role in estimation errors, not affected by the MC simulations, and will also be considered in the evaluation of the nine configurations. Given this paper’s objectives, special attention will be given to comparison between GNSS-assisted and traditional GCP block control.

To present the results, the nine block configurations have been (albeit arbitrarily) ranked according to a decreasing block “strength score” (see Table 6).

The overall calibration accuracy of each block configuration will be measured by the largest residual distortion. To this aim, a grid of 20 by 15 points has been set over the image frame. At each iteration of the MC scheme, the maximum residual distortion error on such grid points (i.e., the distance between the true image distortion correction and the one computed with the estimated distortion parameters) is recorded. Upon completion of MC simulations, the average and standard deviation of the maxima per iteration are computed. To weigh the alternative between GNSS and GCP block control in the calibration, the percentage gain in modelling distortion (reducing the average max residual distortion) will be computed for identical block configurations and similar block control tightness.

### 2.5. Accuracy Evaluation of Ground Coordinates

The accuracy of the ground coordinates in each of the 144 cases is evaluated by comparing, for each tie point coordinate, the true values of the coordinates against the estimated value in each block adjustment. For each check point coordinate, the mean error, the error standard deviation and the RMSE obtained in the 1000 MC iterations is computed and averaged over all the tie points common to all block configurations. As the number of tie points depends on the block configuration, in order for the comparisons to be made on an equal basis, only points common to all configurations have been used in computing the error statistics. As such points are fairly distributed over the survey area, restricting the analysis to the common set doesn’t affect the statistics’ significance. However, this common tie point set has been built excluding the HO configuration (POI case with oblique images only), as very few tie points turned out to be common to other blocks. Using such a small amount, in our opinion, would have affected the significance of the results for all configurations too much. As such, the sample size of the error statistics for HO configuration is not homogeneous with the other configurations [10]. As a matter of fact, in our test the HO case turned out to yield in most cases quite singular results, hardly in agreement with a trend that could be spotted in the other configurations and mainly quite poor [10]. Additionally, in our experiment design we did not think of the POI as a real standalone configuration, but rather as a complement of nadir imagery. A reason for such disappointing results might be that the POI is not that far from an “orbital motion” critical configuration [45]. We present them anyway, with this caveat and without any further comment.

### 2.6. Dome Effect and Pre-Calibration

Our test has not been specifically designed to study the so-called dome effect [22] that may show up when residual calibration errors and weak block geometry produce systematic errors in tie point coordinates, mostly apparent in elevation. Indeed, except in one case (GNSS control case with noGCP), the block control applied in the simulations (see Figure 2) always foresees at least one GCP in the block centre, therefore limiting the magnitude of the Z coordinate error at the block center. However, taking advantage of the 144,000 camera calibration parameter sets estimated in the BBA of the MC simulations in the nine block configurations of the experiment, we investigated the 3D tie point coordinates sensitivity to (inaccurate) pre-calibrated camera parameters, i.e., the dependence of the dome size on the pre-calibration block configuration, through a second MC simulation.

To set “better” conditions for the dome effect to show up, a slightly modified L (ongitudinal) image block configuration has been extracted from the Flat block. In addition to the original tie points, in this block a set of more than 1600 check points has also been generated as follows. The horizontal coordinates of each check point are taken from the nodes of a regular 5 × 5 m grid set over the area, while their elevation is set equal to the average elevation of all the tie points in the original Flat block. The synthetic image coordinates of tie points and check points are then generated error-free, i.e., by projection on the images according to true values of camera parameters, ground coordinates and EO parameters. Finally, only four GCP located at the block corners are used as control in the modified L block.

In the new MC simulation, consisting of 144,000 runs over the modified L block, random errors with a standard deviation of 1 pixel (Medium precision in Table 4) are applied to tie points’ image coordinates only, while check point image coordinates are left unperturbed. The modified L block observations are then adjusted, fixing the four GCP at the corners, in pre-calibration mode, i.e., using fixed camera calibration parameters. Such camera parameters are taken, in each run of the new MC simulation, from one of the 144,000 calibration parameter sets estimated in the first MC simulation. After the BBA, the ground coordinates of the check points are computed by forward intersection (i.e., keeping fixed the estimated EO parameters). In this way, only the effect of the tie point image errors and of the pre-calibrated parameters is transferred via the EO parameters to the check points ground coordinates, as the check points image coordinates are error-free. On completion of the MC simulation then we get 144,000 *Z error* sets for the check points, each set representing the dome effect generated in the flat area by application to the modified L block of a pre-calibrated camera parameter set coming from one of the 144 originally tested configurations.

We divide the 144,000 error sets in 144 groups, according to the block configuration the camera parameters have been estimated on. To summarize the results, for each group (block configuration) the average *Z error*, calculated as the average over all check points of the mean *Z error* of the 1000 MC runs in each check point, is computed. Moreover, the error range due to each pre-calibration configuration is computed as follows. Out of the 1000 runs over each check point, the largest positive, largest negative and standard deviation (being the mean approximately zero for all the points) of the *Z error* are recorded. Finally, the average of all check points differences between the largest positive and negative error (i.e., the maximum range) is computed, hereafter named the *Z error* range. The analysis of both errors should highlight the influence of the pre-calibration block configuration on a dome effect-prone block such as the modified L block.

## 3. Results

In the following, all figures and tables, unless explicitly stated, refer to simulations with random errors generated under Medium precision (see Table 4): 3 cm for CS, 0.5 cm for GCP and 1 pixel for image coordinates.

### 3.1. Camera Calibration Parameters

#### 3.1.1. Principal Distance

**Figure 3:**
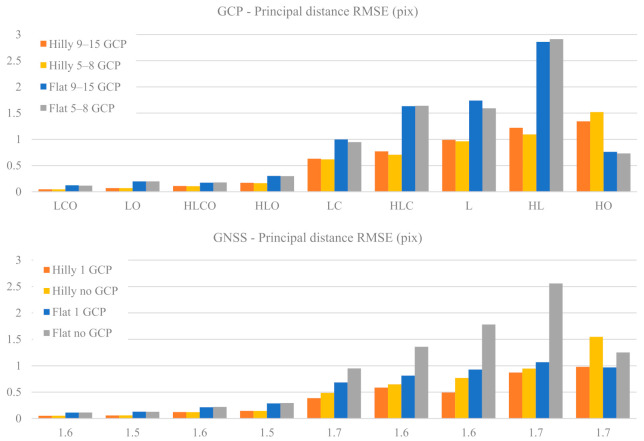
RMSE of the principal distance as a function of block type, terrain type and control tightness: (**top**): GCP case; (**bottom**): GNSS case.

Figure 3 presents the RMSE in pixels of the estimated principal distance (as a function of the nine block configurations, of the terrain type (Flat, Hilly) and of the control tightness (Basic or Enhanced).

The plots show an accuracy deterioration from strong to weak block configurations which is comparatively larger in flat terrain, especially in the GCP case. Oblique images are necessary for accurate estimation of the principal distance: if they are included, the accuracy range is from 0.05 to 0.29 pixel irrespective of control type and tightness as well as terrain type. If they are missing, a sharp decrease in accuracy may occur, from 0.4 to 2.9 pixels. The importance of oblique images becomes apparent when computing the ratio between the principal distance average RMSE of the four LC, HLC, L and HL configurations without oblique images and the corresponding average RMSE of the configurations LCO, LO, HLCO and HLO with oblique images (see Table 7).

As can be seen, the accuracy gap in principal distance determination without and with oblique images ranges from a factor 5 to 9. In the GCP case the gap is largest and almost the same, irrespective of terrain type and control tightness. In the GNSS case, if no GCP is fixed (Basic) the gap is quite significant, while it is the lowest if 1 GCP is fixed (Enhanced), especially in flat terrain. This on the one hand means that, in flat terrain, oblique images are even more necessary than in hilly ones; on the other hand, that GNSS control with 1 GCP partly compensates for a less geometrically strong block configuration. 

Without oblique images, in the GCP case it is the terrain type that ensures (Hilly) or prevents (Flat) accurate determination of the principal distance, while control tightness plays only a minor role; flat terrain is critical also in the GNSS case as, unless a single GCP is employed, the estimation error raises quickly well above 1 pixel. In both GCP and GNSS cases, the same block configurations in hilly terrain provides better results than in a flat one (on average about two times better in our test settings). The HO case (a single ring of oblique images) stands out: it is the only case where, with GCP control, Flat is more precise than Hilly and in the GNSS case Flat Basic (noGCP) is not markedly worse than Flat Enhanced (1 GCP).

#### 3.1.2. Principal Point Location

**Figure 4:**
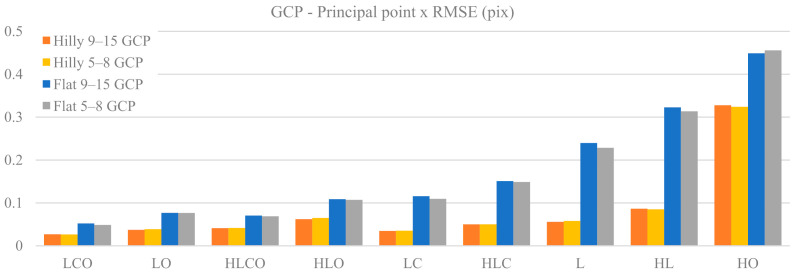

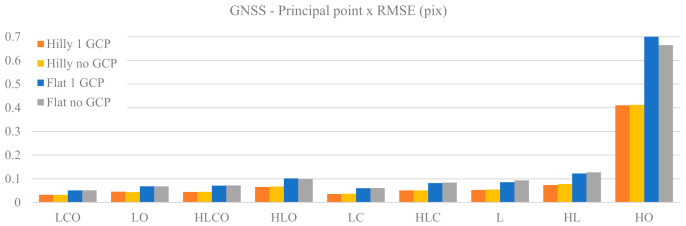
RMSE of the principal point x coordinate as a function of block configuration, terrain type (Flat, Hilly) and control tightness (Basic or Enhanced): (**top**): GCP case; (**bottom**): GNSS case.

**Figure 5:**
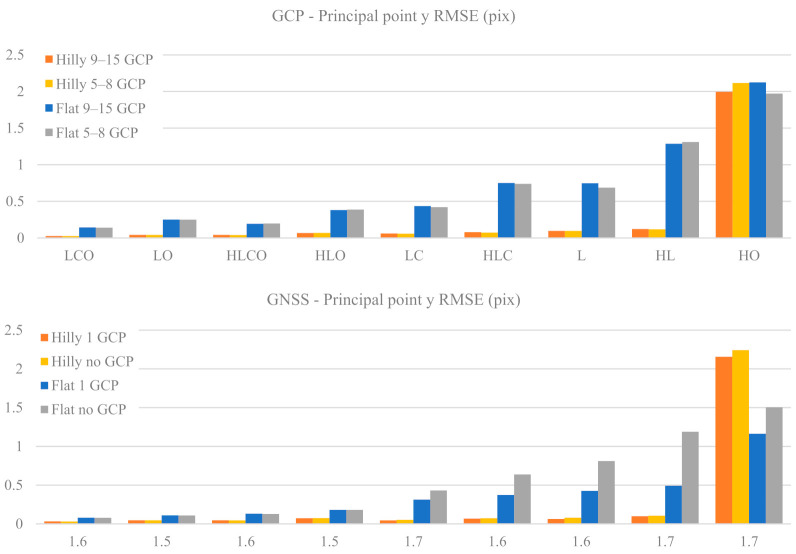
RMSE of the principal point y coordinate as a function of block configuration, terrain type (Flat, Hilly) and control tightness (Basic or Enhanced): (**top**): GCP case; (**bottom**): GNSS case.

Figure 4 and Figure 5 represent the RMSE in pixels of the Principal Point (PP) coordinates PPx and PPy as a function of the nine block configurations, of the terrain type (Flat, Hilly) and of the control tightness (Basic or Enhanced).

With both GCP and GNSS block control, control tightness is not critical for PPx estimation, as the values for Basic and Enhanced cases are very similar. In the GCP case, the accuracy gap between hilly and flat terrain deteriorates markedly moving from strong to weak block configurations, up to a factor 3.8 in HL. To the contrary, in the GNSS case, both for flat and hilly terrain, the accuracy level is weakly dependent on the block configuration and control tightness: indeed, the accuracy gap between the two terrain types is quite stable and never exceeds a factor of 1.7. Finally, the HO case appears again as a singular and critical one, both with GCP or GNSS-assisted block control, and particularly so in the latter case, with an eightfold decrease in accuracy.

PPy accuracy is overall substantially worse than PPx, at least in weak block configurations. In hilly terrain the accuracy gap with respect to PPx is limited for both GCP and GNSS cases: the ratio RMSE_PPx/RMSE_PPy ranges from 1 to 1.7; in flat terrain, to the contrary, the PPy RMSE is worse by a factor ranging from 1.5 (LCO Enhanced) up to 9 (HL Basic).

The plot of the GCP case shows that ground control tightness is not critical for PPy estimation in both terrain types. In the GNSS case, however, this is true only with hilly terrain, while in flat terrain and block configurations lacking oblique images the 1 GCP case is significantly more accurate. In both GCP and GNSS cases and hilly terrain the PPy accuracy is very stable with respect to block configuration and always better than in flat terrain under the same block configuration. In the GNSS case and flat terrain, moreover, the error increases when moving from strong to weak block configurations with a marked jump and at a higher rate when oblique images are removed; a growing gap also opens between Basic (no GCP) and Enhanced (1 GCP) control tightness. The overall relative accuracy gap between the strongest and the weakest block configurations is significant: for the GCP case the error increases by a factor of 3 in hilly terrain to a factor of 9 in flat terrain, while the respective figures for the GNSS case are from 2.4 to 15. Finally, also for PPy, the HO case is critical.

#### 3.1.3. Calibration Overall Accuracy

**Figure 6:**
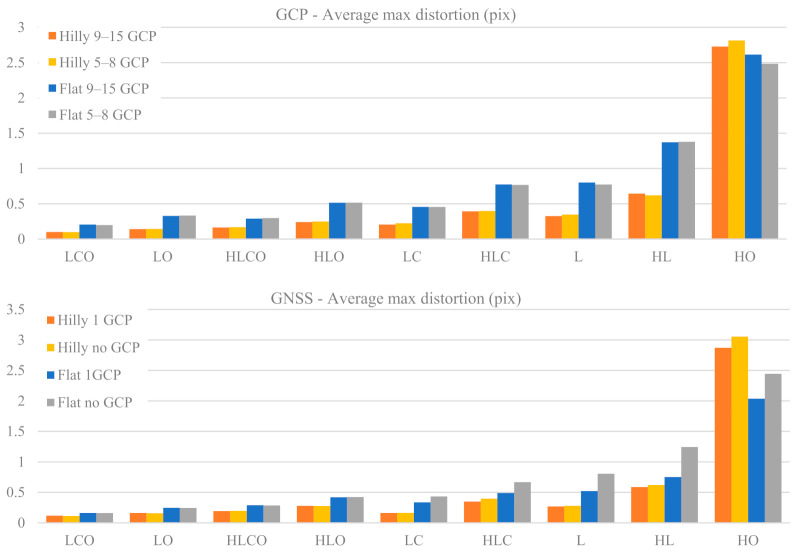
Average value of the maximum distortion error (in pixels) registered over the image frame as a function of the nine cases, of the terrain type (Flat, Hilly) and of the control tightness (Basic or Enhanced), respectively, in the GCP and GNSS cases.

Figure 6 shows the average of the maximum distortion error value registered over the image frame as a function of the nine block configurations, of the terrain type (Flat, Hilly) and of the control tightness (Basic or Enhanced), respectively, in the GCP and GNSS cases.

Both the GCP and GNSS cases show similar trends, with a slight degradation of accuracy for decreasing block configuration strength. A hilly terrain yields more accurate distortion modelling than a flat one: by a factor of 1.8 to 2.3 in the GCP case and from 1.4 to 2.9 in the GNSS case. The HO case is somehow apart, with the largest values about four times worse than the worst result of the other eight block configurations. As far as the block control type is concerned, while in the GCP case the control tightness has little or no influence on distortion accuracy, in the GNSS case with flat terrain the Enhanced control (1 GCP) is clearly more effective when oblique images are missing.

**Figure 7:**
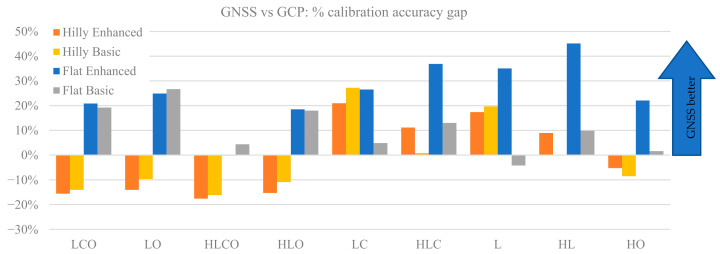
Percentage accuracy gap in camera distortion modelling between GCP and the GNSS case for each of the nine block configurations as a function of the terrain type (Flat, Hilly) and of the control tightness (Basic or Enhanced).

To measure, if any, the overall calibration accuracy gap between the GCP and GNSS case, Figure 7 plots the percentage accuracy gain of performing camera calibration in a GCP or GNSS case, for the nine block configurations. More precisely, for each pair of GCP and GNSS identical configurations, the difference of the average max distortions is computed and expressed as percentage.

${∆}_{maxD}$ of the distortion in the GCP case for each of the nine block configurations, two terrain types and two control cases:

(1) $${∆}_{maxD}=\frac{maxD\left(\mathrm{GCP}\right)-maxD\left(\mathrm{GNSS}\right)}{maxD\left(\mathrm{GCP}\right)}$$

where: $maxD\left(\mathrm{GCP}\right)$ = average value of maximum distortion error over the image frame in the 1000 MC runs when the block configuration is adjusted with the GCP control type. $maxD\left(\mathrm{GNSS}\right)\;$= average value of maximum distortion error over the image frame in the 1000 MC runs when the block configuration is adjusted with the GNSS control type.

In Figure 7 a positive value means the GNSS case is more accurate in modelling the overall image distortion than the GCP case, and vice versa for negative values. Overall, the GNSS delivers a better calibration in most cases, sometimes with quite a significant improvement (up to 45%). In the four strongest block configurations (all with oblique images) GNSS performs markedly better in flat terrain (+23% on average), while GCP is better in hilly terrain (+14% on average). In weaker blocks GNSS performs almost always better than GCP (+20% on average). The largest gains are in flat terrain if at least 1 GCP is used (Enhanced tightness case), with three cases exceeding a 30% gain.

### 3.2. Ground Point Coordinate Accuracy

The ground coordinates accuracy is evaluated by comparing the true against the estimated coordinates for a set of tie points common to all block configurations (see Section 2.5). Such coordinates are influenced by the estimated interior orientation and distortion parameters, whose accuracy, as shown in the previous sections, can vary strongly with the block configuration and control type. At the same time, different block configurations (e.g., LCO vs. HO) have different tie point projections redundancy, projecting ray intersection angles and image multiplicity which affect the accuracy of the tie points as well.

Rather than the magnitude (absolute values) of the coordinates’ RMSE, it seems more appropriate here to present a relative comparison among the different block configurations, as this provides a measure of the accuracy gain when flying according to one or another block configuration. More precisely, the relative accuracy loss Δ_RMSE_ has been computed as:

(2) $${∆}_{\mathrm{RMSE}}=\frac{\mathrm{RMSE}\left(\mathrm{LCO}\right)-\mathrm{RMSE}\left({\mathrm{CFG}}_{i}\right)}{\mathrm{RMSE}\left(\mathrm{LCO}\right)}$$

where RMSE(CFG_i_): average RMSE on tie points in the CFG_i_ configuration, with CFG_i_ = LCO, LO, …, HL and HO; and RMSE(LCO): average RMSE on tie points in the LCO configuration.

**Figure 8:**
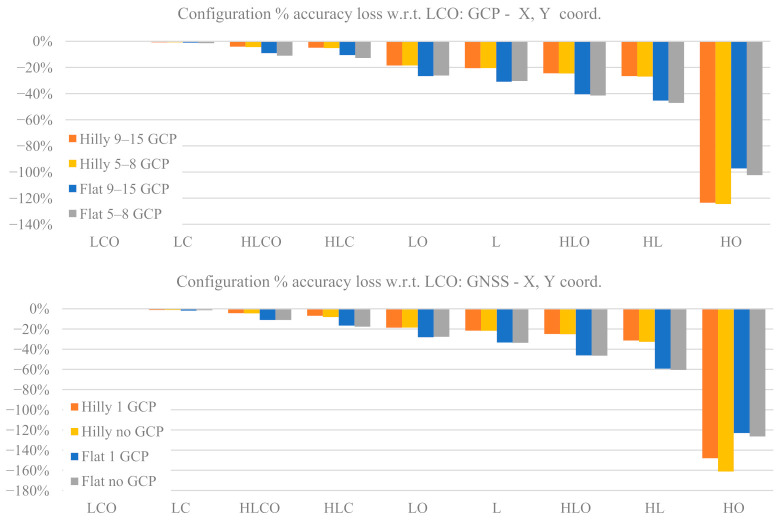

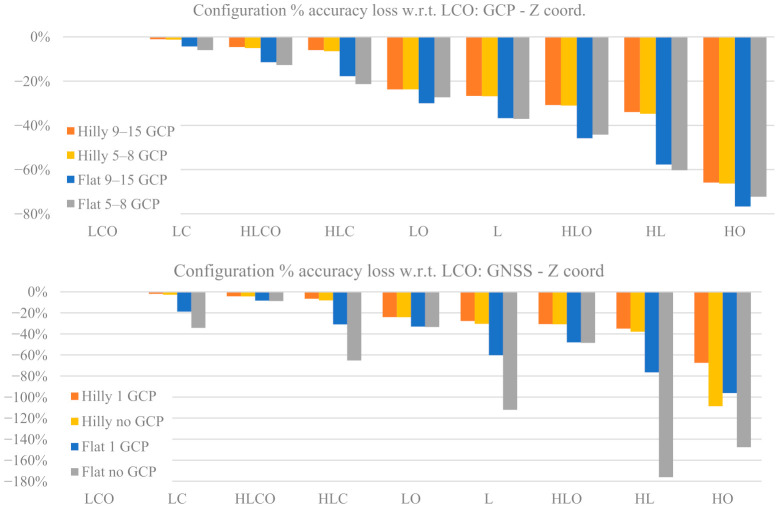
X, Y and Z tie points’ coordinates in GCP and GNSS cases: percentage RMSE loss of each of the nine block configurations w.r.t. the reference block configuration (LCO), as a function of terrain type and control tightness; (**top**): X, Y coordinates; (**bottom**): Z coordinate.

Figure 8 shows the percentage loss Δ_RMSE_ of the ground coordinates RMSE of every block configuration with respect to the reference configuration (LCO) as a function of terrain type and control tightness in the GCP case and in the GNSS case. The top figures refer to horizontal coordinates and the bottom ones to elevation. Please note that the previously used sequence order of the block configuration labels in the graphs has been modified in such a way as to have a monotonic decreasing accuracy.

In both the GCP and the GNSS case, the block configurations split in three groups of similar accuracy: (1) LCO, HLCO, LC and HLC; (2) LO, HLO, L and HL; and (3) HO, which is a singular case. This suggests that cross strips look more important than oblique images to ensure accurate ground coordinates, while the opposite is true for camera calibration parameter estimation accuracy (see Table 7).

For the horizontal coordinates, in the GCP case and hilly terrain, group (1) blocks are roughly equally accurate (differences below 10%); group (2) blocks are 20% to 30% less accurate than LCO; and block HO is 120% less accurate than LCO. In flat terrain the accuracy gap range in group (2) is larger (30% to 50%). In the GNSS case the accuracy gap pattern is basically the same as the GCP case, with a larger group (2) gap (from 35% to 60%).

As far as elevations are concerned, in the GCP case the accuracy gaps in group (2) range from 24% to 35% in hilly terrain and from 35% to 70% in flat terrain. Moreover, in flat terrain a noticeable dependence on control tightness is apparent. A smaller accuracy gap is found in the HO case (from 60% to 80%) with respect to horizontal coordinates. In the GNSS case the picture is more complex. In group (2) the rate of accuracy decrease in flat terrain is larger than in hilly terrain, and even more so between Basic and Enhanced control tightness (the accuracy gap reaches 180%). In HO configuration the accuracy gap goes from 60% (Hilly Dense) to 150% (Flat Sparse).

**Figure 9:**
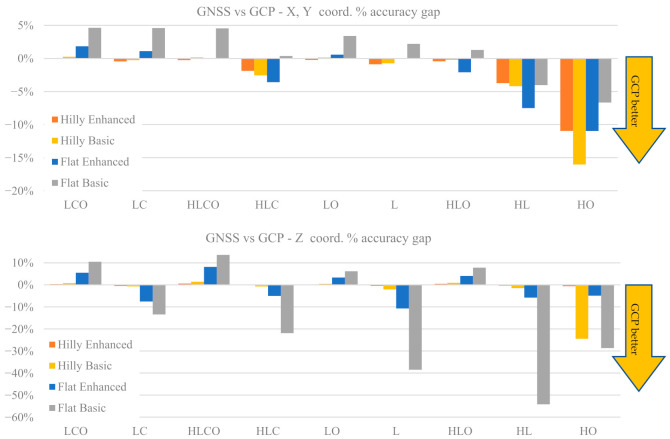
Percentage accuracy gap between the GCP and the GNSS cases, relative to the GCP case, for tie point coordinates’ RMSE as a function of the nine configurations, of the terrain type (Flat, Hilly) and of the control tightness (Basic or Enhanced). Positive values mark a better RMSE for GNSS compared to GCP, and vice versa for negative values. (**Top**): horizontal coordinates; (**bottom**): elevations.

For a comparison between GCP and GNSS case, Figure 9 reports for the tie point coordinates RMSE the percentage gain (or loss) relative to the GCP case. More precisely, the relative accuracy gaps Δ_RMSE_CT_ between GCP and GNSS RMSE for the same configuration have been computed as:

(3) $${∆}_{\mathrm{RMSE}\_\mathrm{CT}}=\frac{{\mathrm{RMSE}}_{\mathrm{GCP}}\left({\mathrm{CFG}}_{i}\right)-{\mathrm{RMSE}}_{\mathrm{GNSS}}\left({\mathrm{CFG}}_{i}\right)}{{\mathrm{RMSE}}_{\mathrm{GCP}}\left({\mathrm{CFG}}_{i}\right)}$$

where: RMSE_GCP_(CFG_i_): average RMSE on tie points in CFG_i_ configuration with GCP block control type; RMSE_GNSS_(CFG_i_): average RMSE on tie points in CFG_i_ configuration with GNSS block control type.

A positive value means the GNSS case is more accurate than the GCP case and vice versa for negative values.

From Figure 9 it can be seen that the horizontal coordinates’ accuracy does not show significant differences between the GCP and GNSS cases: the largest for all configurations (less than 5%) can be expected in flat terrain with basic control; in hilly terrain the differences are below 1%. The HL and HO configurations are (partial) exceptions, with differences up to 8% and 16%, respectively. In elevation the pattern is somehow similar, with differences even more insignificant in hilly terrain. However, in flat terrain there is a clear distinction for block with and without oblique images. In the former case the GNSS case is better (up to 14%) while in the latter the GCP case is markedly better unless the single GCP (Enhanced control case) is fixed: the gap grows from 14% (LC) to almost 55% (HL).

Comparison of GNSS-controlled blocks vs. GCP-controlled ones is strongly influenced by the instruments’ actual precision in Camera Station and GCP coordinate determination and by the weights assigned to such information in the BBA (see Section 2.3). Such precisions, in author’s opinion, are representative of the current state-of-the-art of most UAV surveys. In our test context the two solutions (GCP control network vs. GNSS-assisted orientation) are largely balanced and provide similar tie point accuracy results. Should this not be the case (e.g., should a less-precise on-board receiver be used) one solution would provide significantly better performance than the other.

### 3.3. Effect on Tie Points RMSE of Increased Block Control Precision

**Figure 10:**
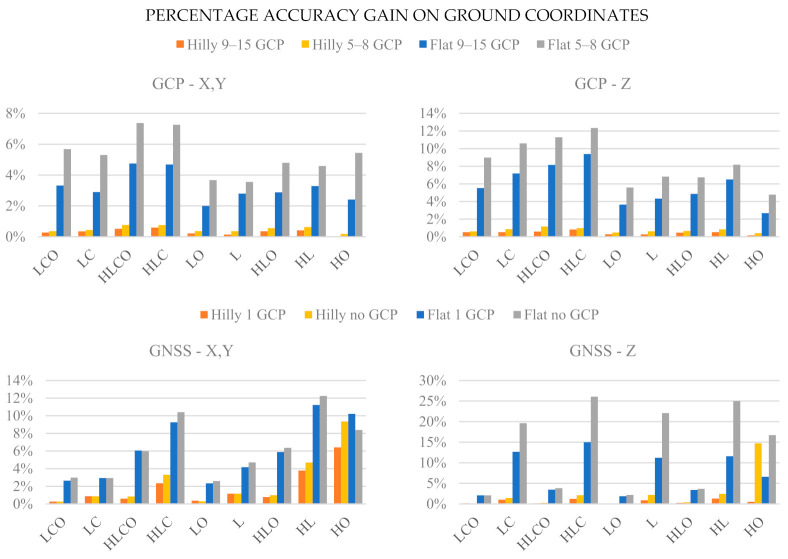
Percentage accuracy gain of the tie points’ ground coordinates when measurement error standard deviations in the MC simulations decrease from 3 cm for CS, 0.5 cm for GCP and 1 pixel for image coordinates (Medium precision in Table 4) to 1 cm, 0.17 cm and 0.33 pixel, respectively (High precision in Table 4). (**Top**): GCP control case; (**bottom**): GNSS control case.

As pointed out at the beginning of this section, all the above-presented results refer to errors in GNSS-determined camera stations, GCP coordinates and image coordinates generated according to Medium precision in Table 4. With error magnitudes three times smaller i.e., generated according to High precision observations as reported in Table 4, the tie points RMSE patterns as a function of block configuration are broadly similar to those shown in the previous paragraph. To measure the improvement (if any) brought by the increased measurement precision, Figure 10 shows the percentage accuracy gain for the tie points ground coordinates achievable with High precision measurements as opposed to Medium precision measurements. More precisely, the accuracy gain Δ_i_ has been computed separately for horizontal (X, Y) and vertical (Z) tie point coordinates as:

(4) $${∆}_{i}=\frac{{\mathrm{RMSE}}_{i}\left(\mathrm{Medium}\right)-{\mathrm{RMSE}}_{i}\left(\mathrm{High}\right)}{{\mathrm{RMSE}}_{i}\left(\mathrm{Medium}\right)}\;i=\left(X,Y\right),Z$$

where ${\mathrm{RMSE}}_{i}\left(\mathrm{Medium}\right)$ = average value of the tie points’ coordinate i RMSE over the 1000 MC runs with observation errors of image coordinates, camera stations and GCP, respectively, of 1 pixel, 3 cm and 0.5 cm. ${\mathrm{RMSE}}_{i}\left(\mathrm{High}\right)\;$= average value of the tie points’ coordinate i RMSE over the 1000 MC runs with observation errors of image coordinates, camera stations and GCP, respectively, of 0.33 pixel, 1 cm and 0.17 cm.

Overall, the ground coordinates’ accuracy increase is very limited in hilly terrain in both the GNSS and GCP cases, where it is lower than 2% in almost all cases, and even less for the horizontal coordinates. In flat areas, accuracy gains, though in absolute terms mostly small, are larger than in hilly terrain in both control cases and, again in both control cases, are more significant for elevations. The gains pattern as a function of the block configuration is, however, different. In the GCP case, perhaps surprisingly, the largest gains (from 5 to 7% in horizontal coordinates and from 6 to 12% in elevation) are found for the stronger configurations with cross strips (LCO, LC, HLCO and HLC). In the GNSS case the only noticeable gains in horizontal coordinates are for the square block (from 6 to 12%). Still in the GNSS case, the largest accuracy gains (from 20% to 26%) are registered for the elevations, in flat terrain and Basic control tightness (no GCP) in block configurations without oblique images.

### 3.4. Dome Effect

As anticipated in Section 2, a second MC simulation has been carried out to evaluate whether and to what extent applying pre-calibrated parameters may still cause the occurrence of the dome effect in the current block being adjusted. In particular, the influence of the pre-calibration block characteristics is investigated. From this new simulation, 144,000 *Z error* sets, each computed on 1600 check points, have been obtained. Every error set represents the dome effect generated on the check points by the application of a pre-calibrated camera parameter set, obtained in one of the first 144,000 MC simulations, in the adjustment of the simulated image observations of a L (ongitudinal) block configuration flown over a flat area, with four GCPs at the corners (see Section 2.6 for details).

**Figure 11:**
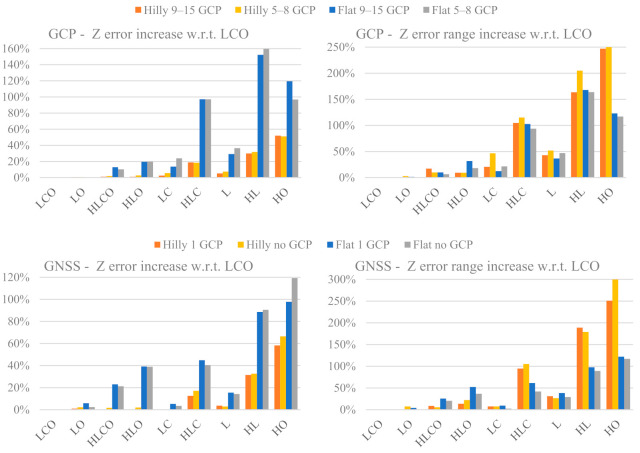
Average *Z error* increment (left) and *Z error* range increment (right) when applying to a L configuration block a pre-calibrated camera parameter set estimated with a given configuration with respect to a pre-calibration set estimated with LCO configuration. The results are presented for both the GCP (**top**) and GNSS (**bottom**) block control cases, as a function of block configuration, block control and terrain type.

The 144,000 error sets have been divided in 144 groups, according to the configuration type of the pre-calibration block. To summarize the results, for each group the average *Z error* and the *Z error* range have been computed. Rather than measuring the magnitude of elevation distortion, here the focus is on the effectiveness of the pre-calibration block configurations in preventing it. Therefore, Figure 11 shows the percentage increase of the average *Z error* and of the *Z error* range of each pre-calibration block configuration with respect to the reference configuration LCO. Both values refer to *Z errors* computed over the 1600 check points in the 1000 adjustments of the modified L (ongitudinal) block configuration with the 1000 camera parameter sets obtained in the former MC simulation. More precisely, the relative percentage differences Δ*_Z_* for the average *Z error* have been computed as:

(5) $${∆}_{Z}=\frac{Z\;error\left({\mathrm{CFG}}_{i}\;pre\_cal.\right)-Z\;error\;\left(\mathrm{LCO}\;pre\_cal.\right)}{Z\;error\;\left(\mathrm{LCO}\;pre\_cal.\right)}$$

where *Z error* (CFG_i_ *pre-cal.*): average *Z error* on 1600 check points in a L block in a flat area adjusted with camera parameters from a pre-calibration block in CFG_i_ configuration, with CFG_i_ = LCO, LO, …, HL and HO; and *Z error* (LCO *pre-cal.*): average *Z error* on 1600 check points in a L block in a flat area adjusted with camera parameters from the reference pre-calibration block. The reference LCO varies according to block control type (GCP or GNSS), control tightness and terrain type.

Likewise, the relative percentage differences Δ_*Z_range*_ for the *Z error* range have been computed as:

(6) $${∆}_{Z\_range}=\frac{eZ\;range\left({\mathrm{CFG}}_{i}\;pre\_cal.\right)-eZ\;range\;\left(\mathrm{LCO}\;pre\_cal.\right)}{eZ\;range\;\left(\mathrm{LCO}\;pre\_cal.\right)}$$

where *eZ range* (CFG_i_ *pre-cal.*): average *Z error* range (difference of the largest positive and the largest negative *Z error*) on 1600 check points in a L block in a flat area adjusted with camera parameters from a pre-calibration block in CFG_i_ configuration, with CFG_i_ = LCO, LO, …, HL and HO; and *eZ range* (LCO *pre-cal.*): average *Z error* range (difference of the largest positive and the largest negative *Z error*) on 1600 check points in a L block in a flat area adjusted with camera parameters from the reference pre-calibration block. The reference LCO varies according to block control type (GCP or GNSS), control tightness and terrain type.

From Figure 11 left, it is apparent that the percentage error gap can be dramatic, especially in flat terrain and in square blocks, if oblique images are missing: the worst case is HL, with 150% and 90% increase in GCP and GNSS case, respectively. In all cases pre-calibration parameters estimated on hilly terrain perform better compared to those on a flat terrain: except for the HO case, the percentage increase of the *Z error* is always less than half compared to that from a calibration over flat terrain, and much less so in the strongest block configurations.

In the GCP case, with pre-calibration executed over a hilly terrain, LO, HLCO and HLO configurations are on par with LCO pre-calibration. This applies also to LC and, perhaps surprisingly, to L (only 7% worse than LCO). Square blocks without oblique images (HLC or HL), on the other hand, deliver calibration parameters that produce *Z errors* 20 to 30% worse. Pre-calibration parameters estimated over a flat terrain with square blocks are not as effective even with oblique images (HLCO +13% and HLO +20%), and much worse without (+97% and +160% in HLC and HL, respectively).

In the GNSS case with pre-calibration executed over hilly terrain, all rectangular configurations (LO, LC and L) and the square configurations with oblique imaging (HLCO and HLO) are on a par with LCO. As in the GCP case, HLC (+17%) and HL (+33%) produce instead significantly larger *Z errors*. In flat terrain a pre-calibration with GNSS rectangular blocks perform better than square ones, as in the GCP case, even if they include oblique images (HLCO +23% and HLO +39%). Comparing GNSS and GCP pre-calibration, GNSS is always better in rectangular blocks and in all square blocks except those including oblique images.

In both the GNSS and the GCP case, the pre-calibration block control tightness seems to play a marginal role (i.e., increasing block control does not significantly reduce the gap with respect to the reference case LCO).

The *Z error* range looks rather independent of the terrain type and block control; it is larger for weaker configurations, but without a clear monotonic trend (i.e., matching the decreasing “block strength” emerging from the previous analysis). The ratio between average height of the dome (Volume/Area) and error range (difference between maximum and minimum height of the dome) is almost constant in flat terrain, from 6 to 7; in hilly terrain instead, it increases from 1.9 to 4.9 with decreasing block strength.

**Figure 12:**
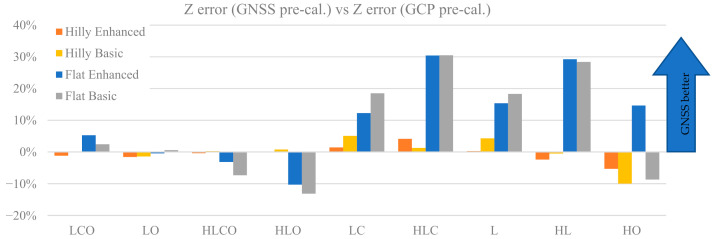
Percentage *Z error* difference on check points for blocks pre-calibrated with camera parameter sets estimated with GNSS-determined camera stations or GCP. Percentages are shown as a function of the nine cases of the terrain type (Flat, Hilly) and of the control tightness (Basic or Enhanced). Positive values mark comparatively smaller *Z errors* for GNSS w.r.t GCP and vice versa for negative values.

A comparison between the effectiveness of camera calibration when taking advantage of GNSS-determined camera stations and when using GCP is among the paper objectives. The average *Z error* obtained using camera parameters from the same calibration block configuration adjusted with GNSS-determined camera stations with respect to the equivalent error obtained from adjustments with camera parameters obtained with GCP control is shown in Figure 12. To compare both pre-calibrations, the percentage difference Δ*_pre-cal_* has been computed as:

(7) $${∆}_{pre-cal}=\frac{Z\;error\left(\mathrm{GCP}\;pre\_cal.\right)-Z\;error\;\left(\mathrm{GNSS}\;pre\_cal.\right)}{Z\;error\;\left(\mathrm{GCP}\;pre\_cal.\right)}$$

Positive Δ*_pre-cal_* values mark comparatively smaller *Z errors* for GNSS pre-calibration w.r.t. GCP pre-calibration and vice versa for negative values.

It is apparent that in hilly terrain both control types are basically equivalent, as differences are below 5%. Likewise, block control tightness is not very important as differences between Basic and Enhanced are also below 5%. In flat terrain with oblique images GCP performs better; however, just slightly so, with differences ranging from almost insignificant (LO, less than 1%) to small (HLO, 13%). Without oblique images, GNSS pre-calibration is better, with improvements up to 30% for square blocks (HLC and HL) and a bit smaller (up to 19%) in rectangular shaped blocks (L and LC). Interestingly, also with Basic tightness (no GCP) the GNSS case seems to deliver better calibration parameters than GCP when flying over a flat terrain.

## 4. Discussion

### 4.1. Camera Calibration Parameters

Overall, as far as the estimation accuracy of the IO parameters is concerned, the GNSS and the GCP cases show similar trends with respect to block configuration, terrain type and block control. Accurate estimation of the principal distance (see Figure 3) is ensured if POI oblique images are included to complement nadir-imaging longitudinal (and possibly cross) strips; if they are missing, the accuracy becomes two-to-five times worse. The HL case in flat terrain is particularly critical for both the GNSS and GCP case (up to ten times worse than the best case). It should also be noted that [10] in a single POI block (HO case) the accuracy is five times worse than the best case. Cross strips, on the other hand, provide only a marginal improvement. Although, at first thought, this might seem surprising, the image block being much more rigid with cross-strips, it is actually in line with findings from [10,18] where cross strips attained less than expected improvements or worse results. It should be noted, as far as principal distance is concerned, that nadir-imaging cross strips do not introduce significant new geometrical constraints (from a projective point of view) for its estimation. On the contrary, having a more significant depth change in the scene pictured by the oblique images (as well as due to the object geometry e.g., as in the hilly study area), drastically increases the accuracy of the estimation.

The accuracy of Principal Point (PP—see Figure 4 and Figure 5) estimation in hilly terrain is very stable with respect to block configuration and control tightness, while in flat terrain the accuracy gets worse with weak block geometries. It should be noted, in this context, that the use of cross strips increases, although not drastically, the determination of the PP location. In fact, it is well known (see for instance [8]) that the use of 90-degree-rolled images in a calibration image block prevents, or at least reduces, the insurgence of unwanted correlations between the parameters and in particular the ones associated to the PP.

The average and standard deviation of the maximum residual distortion affecting the image coordinates after camera calibration parameter estimation (see Figure 6) show trends quite similar to those of PP accuracy estimation. It is worth pointing out that, in this analysis, the distortion error considers both the effects due to a not accurate estimation of the radial and tangential calibration parameters and the ones induced by a not accurate Principal Distance and Principal Point estimation. In other words, the reported errors represent the image coordinates error on image plane due to all the estimated parameters. It is therefore intuitive that this analysis shows similar trends of the ones in Figure 4 and Figure 5. The HO case is a stunning exception in the GNSS case as, even in hilly terrain, the accuracy is more than ten times worse than the best case. This is also true for the maximum average distortion, where HO shows a clear gap compared to other configurations.

To summarize the comparison between block control by GNSS or GCP, there is perhaps no outright winner, but a clear edge for the GNSS case, which performs better especially in weaker block geometry configurations. In agreement with findings from [10,27], accurate determination of all interior orientation parameters is possible with GNSS even without GCP, if oblique images can be included. At first sight, this seems to contradict authors’ [40] and others’ previous tests [34]. However, it should be noted that in both the cited cases the GNSS-assisted blocks were made of nadir images only, as the flights were performed with fixed-wing platforms. Moreover, the authors of [35], flying only longitudinal strips, found adding 1 GCP necessary and sufficient to recover bias in elevation due to inaccurate determination of principal distance.

The question about the optimal survey block configuration is likely to remain open, as the variety of parameters to explore is really too large. As far as our contribution to this point is concerned, a few basic configurations and their combinations have been taken into account. However, some promising variants in the imaging geometry i.e., flying the longitudinal strips with moderate-to-strong (30° to 45°) camera axis inclination along flight direction [26] that recently received attention [10,27,28] were not considered. Another caveat applies to block size and shape, especially in the GNSS case, as pointed out in [27]: should large blocks be composed by juxtaposing basic, optimized sub-block tiles? Do results found with this and other simulations apply to any block size and shape? Is a complete layer of oblique images necessary to complement a basic longitudinal strip layer or, as suggested in [14], is taking just one at each block corner enough? From the results, longitudinal nadir-only blocks should be limited to hilly terrain (in the presented case the largest image scale was three times bigger than the smallest one), where calibration is still fine and the accuracy loss on ground coordinate (see next section) compared to LCO is negligible in horizontal coordinates and does not exceed 20% in elevation. This agrees with [1]. Adding two flight layers (C and O) to the basic longitudinal one delivers of course the top results. It should be noted that, in most cases (see Figure 4, Figure 5 and Figure 6) dropping one of the two results in significant worse accuracies (at least as far as the percentage error increment is considered) of the estimated camera model parameters, but does not result in a significant accuracy loss for the ground coordinates (see Figure 8), except for the GNSS control case on flat terrain. If a choice is to be made between cross and oblique, our results are ambiguous. In flat terrain oblique images are necessary for accurate determination of all IO parameters, while cross strips are only effective with PP coordinate estimation. On the other hand, LC and HLC configurations for rectangular and square blocks show significantly better RMSE on tie point coordinates for hilly terrain, and better or comparable ones for flat terrain compared to LO and HLO.

Do GNSS-based and GCP-based image blocks deliver equivalent calibration accuracy? Broadly speaking the answer is negative, as the former performs always better than the latter in flat terrain if 1 GCP is used, with improvements up to 30%, while the latter is 10% to 20% better with strong block configurations in hilly terrain. It should be noted, however, that from a practical standpoint, GNSS-assisted UAV surveys come with significantly fewer operational constraints than traditional GCP-based ones, especially if the area investigated presents accessibility issues and if total time of operation is critical. The simulations seem to confirm what several of the previously cited authors illustrate in their contributions: the current state of the GNSS technologies implemented in most of the modern RTK UAV systems are already precise enough to implement accurate, and maybe also reliable, GCP-free surveys in most of (if not all) operational conditions. In author’s opinion, acquiring also some GCP (at least one) remains an important requirement nonetheless: as far as the accuracy of the ground points is concerned, introducing at least one GCP might highlight some RTK solution bias and reduce it to some extent. In author’s experience the GNSS UAV navigation solution is sometimes affected by systematic errors, easily masked in a pure GNSS-assisted solution. Additional independent ground control constraints can therefore dramatically increase the survey reliability. At the same time, as the simulations highlighted, including at least one GCP in the GNSS-assisted block might increase significantly (though not drastically) also the quality of the IO and distortion parameters estimation, especially for the weaker image block geometries.

### 4.2. Check Point Coordinates Accuracy

With the exception of HO case, the accuracy loss of horizontal coordinates as a function of the block configuration grows from just 1% (LC) to 30% (HL) in hilly terrain but reaches 60% (HL) in flat terrain. The double grid configurations show the lower loss (Figure 8). The pattern is similar for the GNSS and GCP cases, though in flat terrain the loss rate is more pronounced for the former.

As far as elevations are concerned, in hilly terrain the pattern is similar to horizontal coordinates, though the loss is higher (38% in HL case) in both the GCP and GNSS cases. In flat terrain, however, the GNSS and the GCP show, to the contrary, clear differences. In the former, without oblique images, the accuracy loss is quite sensitive (up to 175% in HL) to afford the lack of ground control. Adding (at least) a single GCP does not really solve the problem as the overall loss remains very high (75% in HL). To the contrary, with inclusion of oblique images, there is no difference between adding or not the single GCP and the overall loss is below 50% in the worst case (HLO). This suggests that adding the GCP as proposed in [40] is not the best solution to error estimation in the principal distance: using a stronger block configuration is more effective. In the light of [27] results and of authors’ findings, a double grid with a moderate pitch angle configuration seems the best trade-off, though perhaps not yet an operational solution for many fixed-wing platforms.

In the GCP case, increasing the control tightness does not bring substantial improvements in horizontal coordinates; in elevations the gains are a bit higher, but not much. Though a meaningful comparison is difficult, this result only partly agrees with findings in [12].

Accuracy gains by increasing by a factor three the control precision (Figure 10) are very limited in hilly terrain, being less than 5% in both horizontal and vertical coordinates. In flat terrain the situation is more complex. In GCP case the improvement is between 5% and 10% in elevation and mostly less than 5% in horizontal coordinates. This point agrees with [12] results. With aerial control, the improvement in horizontal coordinates is still modest, below 10%. In elevation, to the contrary, configurations without oblique images gain from 15% (with a single GCP fixed) to 25% (without GCP) while the remaining are basically not affected.

### 4.3. Dome Effect

Before discussing the results of Section 3.4, it should be stressed again that they refer to the case of pre-calibration only. In other words, what has been presented is an analysis of the pre-calibration block configuration performance in possibly delivering an effective camera calibration parameter set. All the IO and distortion parameter sets evaluated in the different image block configurations were applied (i.e., were used as pre-calibrated parameters) in a single (always the same) L (ongitudinal) image block. For the results presented in Figure 11, the LCO configuration has been taken as “gold standard” and the results of the other configuration types have been measured relative to that case, in order to measure the calibration accuracy loss when pre-calibrating with a weaker block configuration.

As far as *Z error* increase is concerned (see Figure 11 left), a pre-calibration over hilly terrain with both control types (GCP and GNSS) is always better than one over flat terrain. Moreover, except for some weaker configurations (i.e., HLC and HL) the increase in *Z error* is very limited i.e., up to 7% worse. For HLC the increase is ca. 19%, while for HL is stronger (32%). In flat terrain, on the other hand, if the configuration includes oblique imaging, the accuracy loss is minimal only for rectangular block shapes (LO) while in square blocks (HLCO and HLO) the gap is noticeable (10 and 20% in GCP case and 23 to 40% in the GNSS case). Without oblique imaging, there are again similarities between GCP and GNSS control, but the gap loss with respect to LCO is reversed (now GCP is almost twice worse than GNSS). In other words, the weaker the pre-calibration block configuration, the more that an accurate camera station position helps in camera calibration. Again, square blocks are less effective than rectangular ones: LC and L are about three to four times better (GCP case) or even more (GNSS case) than HLC and HL. Motivations for this behaviour should be further investigated. In fact, the differences between the square vs. rectangular image blocks resides only, in authors’ opinion, in a number of observations approximately two times larger, that should not be enough to justify the results. At the same time, analysing the results of camera model parameters estimations and the connected ground point accuracy (see previous sections), even if square (H) blocks provide usually worse results, the differences with rectangular configurations are much smaller.

It is interesting to compare the results presented in Figure 11 with those concerning the actual accuracy in determining the IO and calibration parameters (shown in Figure 3, Figure 4, Figure 5 and Figure 6) and the associated behaviour of the different image block configurations.

Looking at the increase in the *Z error* range (see Figure 11 right) three points can be stressed: the loss with respect to LCO is generally much larger; pre-calibration on hilly terrain does not rule out the chance of large errors; the gap between flat and hilly terrain is mostly small in the GCP case but, in the GNSS case for HLC and HL configurations, the error range for pre-calibration in hilly terrain is even larger than in flat terrain (a fact yet without a clear explanation).

The comparison between GCP-based and GNSS-assisted (camera-based) block control shows that pre-calibration with the latter is generally a better option, as smaller *Z errors* compared to GCP control are obtained. Indeed, results shown in Figure 12 indicate that, as long as oblique imaging is included in the block, it makes little difference in terms of *Z error* whether block control is achieved with GCP or GNSS, as all LCO, LO, HLCO and HLO configurations obtain similar errors with both control types. This can be seen as in agreement with the claim of [13] that calibration is first and foremost a matter of block imaging geometry and camera modelling and that oblique imaging is an essential element of such imaging geometry in blocks flown over flat terrain as well as, generally, with all previous works on optimal imaging for camera calibration. On the other hand, looking at weaker configurations, when imaging geometry is less robust (LC, HLC, L and HL), camera projection centres are more helpful than GCP in flat terrain. There also seems to be a dependence of the improvement amount on the block shape, while cross strips seem less important. Indeed, with our test settings the gain is limited (from 12 to 19%) in rectangular blocks (LC and L), while it is larger (up to 30%) with square blocks (HLC and HL). In short, if, for whatever reason, oblique imaging is not applicable in a survey over flat terrain, using GNSS-assisted orientation is more advisable than using GCP; the remarkable indication is that this applies also when no GCP are available on ground i.e., in the Basic tightness control case, which is in agreement with results shown in Figure 6 and Figure 7.

## 5. Conclusions

Drawing conclusions in a topic as complex as UAV camera calibration with reasonable confidence on their scope and validity is never easy, as the results always come out of given experiment settings, never exhaustive of the multi-dimensional space of the process relevant parameters. As such, keeping in mind the test characteristics depicted in Section 2, a few conclusions are presented in the following.

As far as accuracy of interior orientation parameters is concerned, though trivial to say, the calibration block configuration matters a lot: the accuracy decrease could be as high as 30 times in the worst case for the principal distance, though less (nine times) for the PP coordinates. Oblique images help a lot (LO is almost as good as LCO), though a POI-only (HO) calibration is not recommendable: in our findings nadir looking images are also necessary. The comparison between ground (GCP) and aerial (GNSS-assisted) block control configurations shows that over flat terrain the latter deliver 20% to 60% more accurate calibration parameters than the former, in almost all configurations and for all IO parameters. In hilly terrain GCP control is generally better, though no more than 20%. Unless oblique images are included, estimation of principal distance in the GNSS case over flat terrain might result in large errors.

Estimation errors of the calibration parameters in a pre-calibration block, when applied as fixed parameters in a subsequent BBA, affect ground point coordinates. In this respect our conclusions are that configuration of the pre-calibration block matters in general, and particularly when flying over flat terrain. The average *Z error* increase for weaker configurations compared to LCO can be as large as 150% with GCP control; less so, but still up to 90% for GNSS control. GNSS-assisted block control is in most cases a better option than GCP control in pre-calibration (only with oblique imaging included the difference is minimal). In weaker configurations over flat terrain, camera station positions constrain the block more than GCP.

As far as tie point ground coordinates RMSEs are concerned, weakening the calibration block configuration leads to sizeable but limited percentage accuracy losses in hilly terrain (below 35%) while losses reach 70% in elevation in flat terrain. Block control by GNSS or by GCP are in practice equally accurate in horizontal coordinates, while in elevation GNSS without oblique imaging and no GCP might perform up to 50% worse.

Simulations also confirm that, as many practical experiments have shown, in the GNSS case GCP are generally not necessary for both horizontal coordinates and elevations; however, in flat terrain oblique imaging is necessary to avoid errors in the latter.

## Figures and Tables

**Table 1:** Summary of the real blocks’ characteristics used as a basis for simulated data generation.

Description	Block “Flat”	Block “Hilly”
Area size (Width × Height) (m)	480 × 290	450 × 300
Terrain type	Flat	Hilly
DSM hmin–hmax a.s.l. (m)	136–160 (tree tops)	218–305
# images	396	
# long. strips/# images per strip	7/24	
# cross strips/# images per strip	13/12	
# oblique images rings	2 × 36	
Flight abs. elevation a.s.l. (m)	190	355
Flight elevation a.g.l. (m)	49	49
GSD min–max (cm)	1.3–2.4	2.2–6.0
baselenght (long. & cross) (m)	13	13
# tie points, effective overlap	111,000, 7	85,000, 15
Reprojection error (pix)	0.97	1.59

**Table 2:** Camera parameters estimated in the BBA.

	Principal Distance	Principal Point	Radial Distortion	Decentring Distortion	Scaling and Non-Orthogonality
**Acronym**	c	PPx, PPy	k1, k2, k3	P1, P2	B1, B2

**Table 4:** Measurement precisions levels considered in the simulations.

Precision Level	CS Coord. (cm)	GCP Coord. (cm)	Tie Point Img. Coord. (pix)
Medium	3.0	0.5	1
High	1.0	0.17	0.33

**Table 5:** Parameters accounted for in the simulated BBA.

Parameter	
Area shape and Block type	rectangular: L, LC, LCO, LO
	square: HL, HLC, HLCO, HLO, HO
Landform	Flat, Hilly
Measurement precision	Medium, High
Block control case	GNSS, GCP
Block control tightness	Basic, Enhanced

**Table 6:** Block configurations ranked by decreasing “strength”.

1	2	3	4	5	6	7	8	9
LCO	LO	HLCO	HLO	LC	HLC	L	HL	HO

**Table 7:** Ratio between average principal distance RMSE for LC, HLC, L and HL configurations and average principal distance RMSE for LCO, LO, HLCO and HLO configurations for different terrain types, block control type and tightness.

Control	Enhanced		Basic	
	Hilly	Flat	Hilly	Flat
GNSS	6.1	4.7	7.5	8.7
GCP	9.0	9.1	8.7	9.0

## Data Availability

Data supporting the findings of this study could be available from the authors on request.

## References

[1] Fraser P.C. Camera Calibration Considerations for UAV Photogrammetry Cameras for Drones/UAS/UAVs. Proceedings of the ISPRS Technical Commission II Symposium.

[2] Brown D.C. (1971). Close-Range Camera Calibration. Photogramm. Eng..

[3] Tsai R.Y. (1987). A Versatile Camera Calibration Technique for High-Accuracy 3D Machine Vision Metrology Using Off-the-Shelf TV Cameras and Lenses. IEEE J. Robot. Autom..

[4] Zhang Z. (2000). A Flexible New Technique for Camera Calibration. IEEE Trans. Pattern Anal. Mach. Intell..

[5] Remondino F., Fraser C., Remondino F. (2006). Digital Camera Calibration Methods Considerations and Comparisons digital camera calibration methods: Considerations and comparisons. Int. Arch. Photogramm. Remote Sens. Spat. Inf. Sci..

[6] Fraser C.S. (1982). On the Use of Nonmetric Cameras in Analytical Close-Range Photogrammetry. Can. Surv..

[7] Gruen A., Beyer H.A. (2001). System Calibration Through Self-Calibration. Calibration and Orientation of Cameras in Computer Vision 2001.

[8] Luhmann T., Robson S., Kyle S., Boehm J. (2019). Close-Range Photogrammetry and 3D Imaging.

[9] Clarke T.A., Fryer J.G. (1998). The Development of Camera Calibration Methods and Models. Photogramm. Rec..

[10] Sanz-Ablanedo E., Chandler J.H., Ballesteros-Pérez P., Rodríguez-Pérez J.R. (2020). Reducing Systematic Dome Errors in Digital Elevation Models through Better UAV Flight Design. Earth Surf. Process. Landf..

[11] Cramer M., Przybilla H.J., Zurhorst A. UAV Cameras: Overview and Geometric Calibration Benchmark. Proceedings of the International Archives of the Photogrammetry, Remote Sensing and Spatial Information Sciences—ISPRS Archives, International Conference on Unmanned Aerial Vehicles in Geomatics.

[12] Harwin S., Lucieer A., Osborn J. (2015). The Impact of the Calibration Method on the Accuracy of Point Clouds Derived Using Unmanned Aerial Vehicle Multi-View Stereopsis. Remote Sens..

[13] Carbonneau P.E., Dietrich J.T. (2017). Cost-Effective Non-Metric Photogrammetry from Consumer-Grade SUAS: Implications for Direct Georeferencing of Structure from Motion Photogrammetry. Earth Surf. Process. Landf..

[14] Radford C.R., Bevan G. A Calibration Workflow for “Prosumer” Uav Cameras. Proceedings of the International Archives of the Photogrammetry, Remote Sensing and Spatial Information Sciences—ISPRS Archives, ISPRS Geospatial Week 2019.

[15] Griffiths D., Burningham H. (2019). Comparison of Pre- and Self-Calibrated Camera Calibration Models for UAS-Derived Nadir Imagery for a SfM Application. Prog. Phys. Geogr..

[16] Rosnell T., Honkavaara E. (2012). Point Cloud Generation from Aerial Image Data Acquired by a Quadrocopter Type Micro Unmanned Aerial Vehicle and a Digital Still Camera. Sensors.

[17] Forlani G., Diotri F., Morra Di Cella U., Roncella R. UAV block georeferencing and control by on-board gnss data. Proceedings of the International Archives of the Photogrammetry, Remote Sensing and Spatial Information Sciences—ISPRS Archives, XXIV ISPRS Congress (2020 Edition).

[18] Cramer M., Zhang S. Quality Assessment of High-Resolution UAV Imagery and Products. Proceedings of the 40. Wissenschaftlich-Technische Jahrestagung der DGPF in Stuttgart.

[19] Barazzetti L., Mussio L., Remondino F., Scaioni M. Targetless camera calibration. Proceedings of the ISPRS—International Archives of the Photogrammetry, Trento 2011 Workshop.

[20] Fraser C.S. (2013). Automatic Camera Calibration in Close Range Photogrammetry. Photogramm. Eng. Remote Sens..

[21] Nesbit P.R., Hugenholtz C.H. (2019). Enhancing UAV-SfM 3D Model Accuracy in High-Relief Landscapes by Incorporating Oblique Images. Remote Sens..

[22] Wackrow R., Chandler J.H. (2008). A Convergent Image Configuration for DEM Extraction That Minimises the Systematic Effects Caused by an Inaccurate Lens Model. Photogramm. Rec..

[23] Wackrow R., Chandler J.H. (2011). Minimising Systematic Error Surfaces in Digital Elevation Models Using Oblique Convergent Imagery. Photogramm. Rec..

[24] James M.R., Robson S. (2014). Mitigating Systematic Error in Topographic Models Derived from UAV and Ground-Based Image Networks. Earth Surf. Process. Landf..

[25] Zhou Y., Rupnik E., Meynard C., Thom C., Pierrot-Deseilligny M. (2020). Simulation and Analysis of Photogrammetric UAV Image Blocks-Influence of Camera Calibration Error. Remote Sens..

[26] Taddia Y., Stecchi F., Pellegrinelli A. (2020). Coastal Mapping Using Dji Phantom 4 RTK in Post-Processing Kinematic Mode. Drones.

[27] Stott E., Williams R.D., Hoey T.B. (2020). Ground Control Point Distribution for Accurate Kilometre-Scale Topographic Mapping Using an Rtk-Gnss Unmanned Aerial Vehicle and Sfm Photogrammetry. Drones.

[28] James M.R., Antoniazza G., Robson S., Lane S.N. (2020). Mitigating Systematic Error in Topographic Models for Geomorphic Change Detection: Accuracy, Precision and Considerations beyond off-Nadir Imagery. Earth Surf. Process. Landf..

[29] Sanz-Ablanedo E., Chandler J.H., Rodríguez-Pérez J.R., Ordóñez C. (2018). Accuracy of Unmanned Aerial Vehicle (UAV) and SfM Photogrammetry Survey as a Function of the Number and Location of Ground Control Points Used. Remote Sens..

[30] Tonkin T.N., Midgley N.G. (2016). Ground-Control Networks for Image Based Surface Reconstruction: An Investigation of Optimum Survey Designs Using UAV Derived Imagery and Structure-from-Motion Photogrammetry. Remote Sens..

[31] Friess P. (1989). Empirical Accuracy of Positions Computed from Airborne GPS Data. High Precision Navigation.

[32] Ackermann F., Schade H. (1993). Application of GPS for Aerial Triangulation. Photogramm. Eng. Remote Sens..

[33] Mirjam B., Eija H., Juha J. (1998). GPS supported aerial triangulation using untargeted ground control. Int. Arch. Photogramm. Remote Sens..

[34] Hugenholtz C., Brown O., Walker J., Barchyn T., Nesbit P., Kucharczyk M., Myshak S. (2016). Spatial Accuracy of UAV-Derived Orthoimagery and Topography: Comparing Photogrammetric Models Processed with Direct Geo-Referencing and Ground Control Points. Geomatica.

[35] Zhang H., Aldana-Jague E., Clapuyt F., Wilken F., Vanacker V., van Oost K. (2019). Evaluating the Potential of Post-Processing Kinematic (PPK) Georeferencing for UAV-Based Structure-from-Motion (SfM) Photogrammetry and Surface Change Detection. Earth Surf. Dyn..

[36] Zhou Y., Rupnik E., Faure P.H., Pierrot-Deseilligny M. (2018). GNSS-Assisted Integrated Sensor Orientation with Sensor Pre-Calibration for Accurate Corridor Mapping. Sensors.

[37] Forlani G., Diotri F., di Cella U.M., Roncella R. (2019). Indirect UAV Strip Georeferencing by On-Board GNSS Data under Poor Satellite Coverage. Remote Sens..

[38] Peppa M.V., Hall J., Goodyear J., Mills J.P. Photogrammetric Assessment and Comparison of Dji Phantom 4 pro and Phantom 4 Rtk Small Unmanned Aircraft Systems. Proceedings of the International Archives of the Photogrammetry, Remote Sensing and Spatial Information Sciences—ISPRS Archives, ISPRS Geospatial Week 2019.

[39] James M.R., Robson S., Smith M.W. (2017). 3-D Uncertainty-Based Topographic Change Detection with Structure-from-Motion Photogrammetry: Precision Maps for Ground Control and Directly Georeferenced Surveys. Earth Surf. Process. Landf..

[40] Benassi F., Dall’Asta E., Diotri F., Forlani G., Cella U.M., Roncella R., Santise M. (2017). Testing Accuracy and Repeatability of UAV Blocks Oriented with Gnss-Supported Aerial Triangulation. Remote Sens..

[41] James M.R., Robson S. (2012). Straightforward Reconstruction of 3D Surfaces and Topography with a Camera: Accuracy and Geoscience Application. J. Geophys. Res. Earth Surf..

[42] Dall’Asta E., Delaloye R., Diotri F., Forlani G., Fornari M., di Cella U.M., Pogliotti P., Roncella R., Santise M. Use of Uas in a High Mountain Landscape: The Case of Gran Sommetta Rock Glacier (AO). Proceedings of the International Archives of the Photogrammetry, Remote Sensing and Spatial Information Sciences—ISPRS Archives, ISPRS Geospatial Week 2015.

[43] Roncella R., Forlani G., Diotri F. (2021). A monte carlo simulation study on the dome effect. Int. Arch. Photogramm. Remote Sens. Spat. Inf. Sci..

[44] James M.R., Robson S., d’Oleire-Oltmanns S., Niethammer U. (2017). Optimising UAV Topographic Surveys Processed with Structure-from-Motion: Ground Control Quality, Quantity and Bundle Adjustment. Geomorphology.

[45] Sturm P. Critical Motion Sequences for Monocular Self-Calibration and Uncalibrated Euclidean Reconstruction. Proceedings of the IEEE Computer Society Conference on Computer Vision and Pattern Recognition.

